# Supplementary material for: Content-rich biological network constructed by mining PubMed abstracts
Source: BMC Bioinformatics. 2004 Oct 8;5:147. doi: 10.1186/1471-2105-5-147 (PMC528731; doi:10.1186/1471-2105-5-147)
Supplement: Additional File 2 — The original results of the above study (non-essential files are deleted to keep the file size under the limit set by BMC bioinformatics). [file 1471-2105-5-147-S2.bz2 › chilibotAdditionalFile2/dip10/297ID7486683E1288/html/PIK3R1_IRS1.html]

 


 **PIK3R1** and **IRS1** 
  
Found 143 abstracts in PubMed, retrieved 10.  
 

 What does Google say? 
 PDF only 
| .edu only 

---

**Interactive relationship** (e.g. stimulation, inhibition, etc)

**Stimulatory relationship**- Exposure to IGF 1 for 30 minutes or E2 for 20 minutes increased insulin receptor substrate 1  [ **IRS1** ]  IRS 1  [ **IRS1** ]  association with the regulatory p85 subunit of PI3K  [ **PIK3R1** ] , enhanced tyrosine phosphorylation of p85, and increased PI3K  [ **PIK3R1** ]  activity.  Ref: 12701063 Metabolism, 2003
- In contrast, there was a high level of IRS  [ **IRS1** ]  2 expression and insulin stimulated tyrosyl phosphorylation as early as embryonic day 15 with robust PI3K  [ **PIK3R1** ]  binding and activation, which may enhance hepatocyte survival during the rapid growth phase of the liver.  Ref: 12891559 Gastroenterology, 2003
- IRS 1  [ **IRS1** ]  over expression in transgenic Tg mouse livers caused constitutive activation of Erk mitogen activated protein kinase MAPK and phosphatidylinositol 3 kinase PI3K  [ **PIK3R1** ]  resulting in significantly increased levels of DNA synthesis and larger hepatic masses relative to non transgenic non Tg littermates.  Ref: 12763374 J Hepatol, 2003
**Neutral relationship**

**Non-interactive relationship** (e.g. studied together, co-existance, homology, etc.)

- Immunoprecipitation Western blot studies revealed that CLA inhibited IGF I induced phosphorylation of IGF IR and insulin receptor substrate IRS 1  [ **IRS1** ] , recruitment of the p85 regulatory subunit of phosphatidylinositol 3 kinase PI3K  [ **PIK3R1** ]  to IGF IR, IGF IR associated PI3K  [ **PIK3R1** ]  activity, and phosphorylated Akt and extracellular signal regulated kinase ERK 1 2 levels.  Ref: 12888657 J NutrJ Nutr, 2003
- METHODS We determined the expression and tyrosyl phosphorylation of the insulin receptor beta subunit IRbeta, IRS 1  [ **IRS1** ]  and IRS  [ **IRS1** ]  2, the binding of phosphatidylinositol 3 kinase PI3K  [ **PIK3R1** ] , and activation of the mitogen activated protein kinase MAPK pathway in the presence or absence of insulin stimulation in vivo during development and in the adult liver.  Ref: 12891559 Gastroenterology, 2003
- Leptin stimulation activates JAK STAT, IRS 1  [ **IRS1** ]  PI3K  [ **PIK3R1** ]  and MAPK signalling pathways.  Ref: 12823272 Clin Exp Immunol, 2003
- We then identified an increase in JAK2,  **IRS1**  and IRS2 tyrosine phosphorylation and in their association with p85, a subunit of PI3K  [ **PIK3R1** ] .  Ref: 12706299 Mol Cell Endocrinol, 2003
- These data emphasize the importance of IRS  [ **IRS1** ]  2 PI3K  [ **PIK3R1** ]  PKB signal transduction for beta cell growth and survival.  Ref: 12850284 Mol Cell Endocrinol, 2003
- Insulin stimulated insulin receptor substrate IRS 1  [ **IRS1** ]  tyrosine phosphorylation and PI3K  [ **PIK3R1** ]  activity are impaired 40 50% in diabetic subjects compared with lean or obese subjects.  Ref: 12882908 DiabetesDiabetes, 2003
- However, increased IRS  [ **IRS1** ]  3 expression blocked glucose IGF 1 induced IRS  [ **IRS1** ]  2 translocation from the cytosol to the plasma membrane, dampening IRS  [ **IRS1** ]  2 IGF 1R interaction and subsequent activation of the PI3K  [ **PIK3R1** ]  PKB GSK3 signaling pathway.  Ref: 12850284 Mol Cell Endocrinol, 2003
- At the molecular level, impaired activation of the IR IRS  [ **IRS1** ]  PI3K  [ **PIK3R1** ]  pathway and a significant decrease both in the levels and in insulin stimulated activation of the serine threonine kinase Akt were observed.  Ref: 12767044 J Cell Physiol, 2003
- Insulin is a potent inducer of adipogenesis, and differentiation of adipocytes requires many components of the insulin signaling pathway, including the insulin receptor substrate IRS 1  [ **IRS1** ]  and phosphatidylinositol 3 kinase PI3K  [ **PIK3R1** ] .  Ref: 12807888 J Biol Chem< MedlineTA>J Biol Chem, 2003
- Importantly, weight loss in obese subjects normalizes PKClambda zeta activation and increases IRS 1  [ **IRS1** ]  phosphorylation and PI3K  [ **PIK3R1** ]  activity.  Ref: 12882908 DiabetesDiabetes, 2003
